# Supplementary material for: Patterns of Intron Gain and Loss in Fungi
Source: PLoS Biol. 2004 Nov 30;2(12):e422. doi: 10.1371/journal.pbio.0020422 (PMC532390; doi:10.1371/journal.pbio.0020422)
Supplement: Table S1 — Also available at http://genes.mit.edu/NielsenEtAl/. (4.3 MB ZIP). [file pbio.0020422.st001.zip › NielsenEtAl/html/899.html]

AN5134.1.NCU01744.1.MG07187.1.FG01433.1


```
 CLUSTAL W (1.82) Multiple Sequence Alignments - Introns Inserted


Sequence 1: MG07187.1	2118 aa
Sequence 2: FG01433.1	2113 aa
Sequence 3: NCU01744.1	2116 aa
Sequence 4: AN5134.1	2144 aa
Alignment Length: 2152 aa
Number Identitical Residues: 1566 aa
Alignment Score (without introns) 68451


MG07187.1 	MGLTADFDDRQTHIEAESNEYHPYEYQTENNSSWAGALPVKQ2GLYDPDLEKDACGVGFA
NCU01744.1	MGAIEDFDDRQVHLETEKEQYHPYEYQTENNSSWAGALPVKQ2GLYDPELEKDACGVGFA
FG01433.1 	MGLNEDFDDRQIQTEAEQQPYVPYEYQTENNDSWAGALPVKQ~GLYDPSYEKDACGVGFA
AN5134.1  	MGLNLEEIYGQTIVE-EQRPNEYSEYQPKKGYGWANTLPERQ~GLYDPEYEKDACGVGFA
          	**   :    *   * *..     ***.::. .**.:** :* *****. **********

MG07187.1 	C2HIKGKPSHKIVSDA1RNLLCNMTHRGAVGSDARDGDGAGVMTSIPHKFFIKNFEREVD
NCU01744.1	C2HIKGKPSHKIVSDA~RNLLCNMTHRGAVGSDARDGDGAGVMTSIPHKFFVKNFARDVG
FG01433.1 	C2HIKGKPSHKIVSDA~RNLLCNMTHRGAVGSDARDGDGAGVMTSIPHRFFIKNFEKEED
AN5134.1  	A2NIKGKASHKIVSDA1RNLLCNMTHRGAVGSDARDGDGAGVMTSIPHKFFIKNFAREVG
          	. :****.******** *******************************:**:*** :: .

MG07187.1 	IKLPPLGQYAVGNLFFKPDQETLHESKRQLEDIAESLGLRVLGWREPPVDSTLLGPAAKS
NCU01744.1	VTLPPLGQYAVGNLFFKPDPETLQESKRQLEEIAESLGLRVLGWREPPVDSTLLGPAALS
FG01433.1 	IKLPPLGQYAVGNLFFKPDEETLQESKRQLEDVAESLGLRVLGWRRPPVDSTLLGPAAKS
AN5134.1  	VDLPPLGQYAVGNLFFKPDEEALKESIKQFEEIATSLGLRVLGWREVPRDSTILGPAALS
          	: ***************** *:*:** :*:*::* **********. * ***:***** *

MG07187.1 	REPIIMQPFVVLTSAYGAGNAPETTDPEKFDERLFERQLYVLRKRATHTIGLQNWFYLCS
NCU01744.1	REPSILQPFVVLASAYGPGNEPETTDPEKFDERLFERQLYVLRKRATHTIGLQNWFYICS
FG01433.1 	REPIIAQPFVVLASAYGTGNAPEMTDPEKFDERLFERQLYILRKRATQSIGLHNWFYLCS
AN5134.1  	REPTILQPFVVLKSAYGEGNKPDITDPEQFDTKTFELQLYVLRKRATHIIGLANWFYLCS
          	*** * ****** **** ** *: ****:** : ** ***:******: *** ****:**

MG07187.1 	LSNKNIVYKGQLAPVQVYEYYHDLVNADYEAHFALVHSRFSTNTFPSWDRAQPLRWAAHN
NCU01744.1	LSNKNIVYKGQLAPIQVYQYYNDLVNYDYEAHFALVHSRFSTNTFPSWDRAQPLRWAAHN
FG01433.1 	LSNKNIVYKGQLAPVQVYSYYHDLVNADYEAHFALVHSRFSTNTFPSWDRAQPLRWAAHN
AN5134.1  	LSNRNIVYKGQLAPIQVYQYYHDLVNVDYEGHFALVHSRFSTNTFPSWDRAQPLRWAAHN
          	***:**********:***.**:**** ***.*****************************

MG07187.1 	G1EINTLRGNKNWMRAREGVMQSDIFGDELELLYPIVEDGGSDSAAFDNVLELLTINGVL
NCU01744.1	G1EINTLRGNKNWMRAREGVMQSDVFGDELELLYPIVEAGGSDSAAFDNVLELLTINGVL
FG01433.1 	G~EINTLRGNKNWMRAREGVMQSDVFKDELEQMYPVVEDGGSDSAAFDNVLELLTINGVL
AN5134.1  	G~EINTLRGNKNWMRAREGLLKSDIFGEELESLLPIVEDGGSDSAAFDNVLELLMINGVL
          	* *****************:::**:* :*** : *:** *************** *****

MG07187.1 	SLPEAVMLMVPEAWQGNDHMDPKKAAFYEWAACQMEAWDGPALFTFADGRFCGANLDRNG
NCU01744.1	SLPEAVMLMVPEAWQGNQSMDPKKAAFYEWAACQMEPWDGPALFTFADGRFCGANLDRNG
FG01433.1 	SLPEAVMLMVPEAWQGNQHMDPKKAAFYEWAACQMEPWDGPALFTFADGRYCGANLDRNG
AN5134.1  	SLPEAVMIMIPEAWQDNPAMDPAKAAFYEWAACQMEPWDGPALFTFSDGRYCGANLDRNG
          	*******:*:*****.*  *** *************.*********:***:*********

MG07187.1 	LRPCRFYVMDDDRIICASEVGTIPVDPERVIQKGRLQPGKMLLVDTVAGRIIDDSELKAA
NCU01744.1	LRPCRYYVMDDDRIICASEVGTIPVEPEKIIQKGRLQPGRMLLVDTRAGRIIDDSELKAA
FG01433.1 	LRPCRFYVMDDDRIICASEVGTIPVEPESVIQKGRLQPGRMLLVDTQAGRIIDDKELKEA
AN5134.1  	LRPCRFYVTDDDRIICASEVGAVDIDQERVVQKGRLQPGKMLLVDTVAGRIIDDSELKYT
          	*****:** ************:: :: * ::********:****** *******.*** :

MG07187.1 	VSSRQDFRSWLDKELISLPTLVESVAQDKTVQLEAKPDATALQEDPLLHAFGYTFEQVSL
NCU01744.1	VSSREDFRSWLDNELITMPQVLETLSGSQSIELAAKPDNTKIQEDPLLHAFGYTFEQVSL
FG01433.1 	VSSRYDFRAWLDSELITLPKVVEIMER--ALDLAPKLDDKTIQSDPLLHSYGYTHEQVSL
AN5134.1  	VAHRHDFSSWLNKELVKLPAITEKLVEQ-NMDLRHDLDNTTVQNDPRLKAFGYSFEQVTL
          	*: * ** :**:.**:.:* : * :  .  ::*  . * . :*.** *:::**:.***:*

MG07187.1 	LLAPMAQDEKEALGSMGNDSPLACLSQAPHLLYEYFRQLFAQVTNPPIDPIRESVVMSLE
NCU01744.1	LLGPMASDEKEALGSMGNDAPLACLSDAPKLLYEYFRQLFAQVTNPPIDPIRESIVMSLE
FG01433.1 	LLAPMAADEKEALGSMGNDAPLACLTQSPRLLYDYFRQLFAQVTNPPIDPIRESIVMSLE
AN5134.1  	LLGPMGADSKEALGSMGNDAPLACIAKQPRLLYEYFRQLFAQVTNPPIDPIREAVVMSLE
          	**.**. *.**********:****::. *:***:*******************::*****

MG07187.1 	CYVGPQGNLLEMDASQCGRLLLPSPILSIPEFNAIKNMPKLHPEWTVKVIDITFPKKEGV
NCU01744.1	CYVGPQGNLLEMDSSQCGRLLLPSPILSIPEFNALKNMSTIHPEWTVKTIDLTFPKKEGI
FG01433.1 	CYVGPQGNLLEMDASQCGRLLLPSPVLSIEEFNAIKNMSNKYSEWTVKTIDLTFPKNQGV
AN5134.1  	CYVGPQGNLLEMDPSQCRRLLLPSPILSIPEFNALKNINTVHKDWTVRLIDITFEKKKGV
          	*************.*** *******:*** ****:**: . : :***: **:** *::*:

MG07187.1 	EGYMRHLDYICNETTDAIEKRNRVIVLSDRNTSADRVAVSALLASGMVHHHLVSNKWRSM
NCU01744.1	EGYIRHLDYICMEATAAIEASDRIIVLSDRNTSKDRVAVSALLAGGMVHHHLVSNKWRAM
FG01433.1 	QGYIKHLDEICNEASAAIEARDRVIILSDRNTSADRVPVSAVLASAMVHHHLVSNKWRSM
AN5134.1  	PGYIEALDRICDAATEAIQQGDKILILSDRATSADRVPVSALLATGLVHHHLVRNKWRSL
          	 **:. ** **  :: **:  :::::**** ** ***.***:** .:****** ****::

MG07187.1 	AALVVETAEAREVHHMCVLLGYGADAVNPYLAMECILKLNREKLIKKQLTDDMLIYNYKH
NCU01744.1	AAIVAETAEAREVHHMCVLLGYGVDAINPYLAMECIIKLNKEKLIKKKLTDDELIYNYKH
FG01433.1 	AALVVETAEAREVHHMCVLLGYGADAVNPYLAMECILKLNREGLIKKKTTNETLIRNYKH
AN5134.1  	AALIVETAEAREVHHMCVLVGYGADGINPYLAMECILKMNREKLIRKELSDEKVIENYKA
          	**::.**************:***.*.:*********:*:*:* **:*: ::: :* *** 

MG07187.1 	SCDGGILKVMSKMGISTLASYKGAQIFEALGVDDSVVERCFRGTASRIKGVTFETIAEDA
NCU01744.1	SCDGGILKVMSKMGISTLASYKGAQIFEALGVDDSVVERCFKGTASRIKGITFELIAEDA
FG01433.1 	SCDGGILKVMSKMGISTLASYKGAQIFEILGLDETVVERCFRGTASRIQGMTFELIAEDA
AN5134.1  	SCDGGILKVMSKMGISTLASYKGAQIFEALGIDDSVIDRCFTGTASRIRGMNFELIAQDA
          	**************************** **:*::*::*** ******:*:.** **:**

MG07187.1 	FRFHERGFPSRHTVAVPGLPESGEYHWRDGGEPHINDPTSIANIQDAVRTKNDKSYEAYS
NCU01744.1	FRLHERGFPSRYTVAVPGLPESGEYHWRDNGEAHVNDPTSIANIQDAVRTKNDKSYEAYS
FG01433.1 	FRFHERGFPSRASIGPPGLPESGEYHWRDGGEPHVNDPTSIANIQDAVRTKNDKSYEAYS
AN5134.1  	FAIHERGYPSRAIVDIPGLNESGEYHWRDGGEDHINDPVSIANIQDAVRTKNDKSYEAYA
          	* :****:***  :  *** *********.** *:***.********************:

MG07187.1 	RSEYEQIKACTLRGMLDFSFEEATPVPIDQVEPWTEIVRRFCTGAMSYGSISMESHSTLA
NCU01744.1	LSEYEQIKSCTLRGMLDFNFEDCSPIPIDQVEPWTEIVRRFCTGAMSYGSISLESHSTLA
FG01433.1 	RSEYEQIKNCTLRGLLDFKFEDCTPVPIDQVEPWTDIVRRFCTGAMSYGSISMESHSTLA
AN5134.1  	KAAHEQIKNCTLRGMLEFDFDQRTPIPIDQVEPWTEIVRRFVTGAMSYGSISMESHSTLA
          	 : :**** *****:*:*.*:: :*:*********:***** **********:*******

MG07187.1 	VAMNRLGGKSNTGEGGEDPERSQRMPNGDTMRSAIKQVASGRFGVTSAYLADSDELQIKM
NCU01744.1	VAMNRLGGKSNTGEGGEDPERSQRLPNGDTMRSAIKQVASGRFGVTSAYLADSDELQIKM
FG01433.1 	VAMNRLGGKSNTGEGGEDPERSQRMPNGDTMRSAIKQVASGRFGVTSAYLADSDELQIKM
AN5134.1  	VAMNRLGGKSNTGEGGEDPERSKRMENGDTMRSAIKQIASGRFGVTSHYLADADELQIKM
          	**********************:*: ***********:********* ****:*******

MG07187.1 	AQGAKPGEGGELPGHKVSKSIARTRHSTPGVGLISPPPHHDIYSIEDLKQLIYDLKCSSP
NCU01744.1	AQGAKPGEGGELPGHKVSKSIARTRHSTPGVGLISPPPHHDIYSIEDLKQLIYDLKCSSP
FG01433.1 	AQGAKPGEGGELPGHKVSKSIARTRHSTPGVGLISPPPHHDIYSIEDLKQLIYDLKCSSP
AN5134.1  	AQGAKPGEGGELPGHKVVGPIAHTRYSTPGVGLISPPPHHDIYSIEDLKQLIYDLKCSNP
          	*****************  .**:**:********************************.*

MG07187.1 	RSRVSVKLVSETGVGIVASGVAKAKADHILISGHDGGTGASRWTGIKYAGLPWELGLAET
NCU01744.1	RSRVSVKLVSETGVGIVASGVAKAKADHILISGHDGGTGASRWTGIKYAGLPWELGLAET
FG01433.1 	RSRVSVKLVSEVGVGIVASGVAKAKADHILISGHDGGTGASRWTGIKYAGLPWELGLAET
AN5134.1  	RARVSVKLVSEVGVGIVASGVAKAKADHILISGHDGGTGASRWTGIKYAGLPWELGLAET
          	*:*********.************************************************

MG07187.1 	HQTLVLNDLRGRVVVQTDGQLRTGRDVAIACLLGAEEWGFATTPLIAMGCIMMR------
NCU01744.1	HQTLVLNDLRGRVVVQTDGQLRTGRDVAIACLLGAEEWGFATTPLIAMGCIMMR------
FG01433.1 	HQTLVLNDLRGRVVVQTDGQLKTGRDVALACLLGAEEWGFATAPLIAMGCVFMR------
AN5134.1  	HQTLVLNDLRGRVIVQTDGQIRTGRDVAVACLLGAEEFGFATTPLIAMGCIMMSKDPCPV
          	*************:******::******:********:****:*******::* ..... 

MG07187.1 	------------KCHL1NTCP~VGIATQDPELRKKFKGTPEHVINFFYYIANELRAIMAK
NCU01744.1	------------KCHL~NTCP~VGIATQDPELRKKFTGTPEHVINFFYYVANELRAIMAR
FG01433.1 	------------KCHL~NTCP~VGIATQDPELRKKFTGTPEHVINFFYYVANELRAIMAQ
AN5134.1  	YVSLQPLIWCIGKCHL~NTCP0VGIATQDPELRKKFEGQPEHVINFFYYIANELRAIMAK
          	  : ..   . .**** **** ************** * **********:*********:

MG07187.1 	LGFRTINEMVGHAEMLKVRDDLRTNKTENIDLSLILTPAHKLRPGVATFNVRKQDHRLYV
NCU01744.1	LGFRTINEMVGHVEVLKVREDLRTKKTSNIDLSLILTPAHKLRPGVATFNVRKQDHKLYV
FG01433.1 	LGFRTINEMVGHVEVLKMRDDLRTNKTANIDLSLLLTPAHKLRPGVATFNVRKQDHKLHV
AN5134.1  	LGIRTINEMVGRAELLKVRDDLTNPKQENIDLSLILTPAHSLRPGVATYNVRKQDHRLHT
          	**:********:.*:**:*:** . *  ******:*****.*******:*******:*:.

MG07187.1 	RMDNKLISEAELTLDKGLPSRIECDVVNTDRALGTSLSYQISKKYGEAGLPMDTVHVNIK
NCU01744.1	RLDNKLITEAELTLDKGLPSRIECDIVNTDRAMGTSLSYQISKRYGEKGLPDDTVHVNIK
FG01433.1 	RLDNKLISESELTLDKGLPSRIECDIVNTDRAMGTSLSYHISKRYGEAGLPMDTVHVNIK
AN5134.1  	RLDNKLIAESELALEKGLPCRVECDVVNTDRALGATLSYQVSRRYGGEGLPQDTIHSNIK
          	*:*****:*:**:*:****.*:***:******:*::***::*::**  *** **:* ***

MG07187.1 	GSAGQSFGAFLAPGVTLELEGDANDYVGKGLSGGRLVIYPPRSAVFKAEENVLIGNTCLY
NCU01744.1	GSAGQSFGAFLAPGVTLELEGDANDYVGKGLSGGRLIVYPPRSAVFKAEENIIVGNVCLY
FG01433.1 	GSAGQSFGAFLAPGVTLELEGDSNDYVGKGLSGGRLIIYPPRSAVFKSEENILIGNTCLY
AN5134.1  	GSAGQSFGAYLAPGITLELEGDANDYVGKGLSGGRLIVYPPRGAAFKAEENVIVGNTCLY
          	*********:****:*******:*************::****.*.**:***:::**.***

MG07187.1 	GATSGSCFFRGVAAERFAVRNSGATAVVEGVGDHGCEYMTGGRVVVLGSTGRNFAAGMSG
NCU01744.1	GATSGTCFFRGVAAERFAVRNSGATAVVEGVGDHGCEYMTGGRVVILGSTGRNFAAGMSG
FG01433.1 	GATTGTCFFRGVAAERFAVRNSGATAVVEGVGDHGCEYMTGGRVVVLGSTGRNFAAGMSG
AN5134.1  	GATRGTCFFRGVAAERFAVRNSGATAVVEGVGDHGCEYMTGGRVVVLGSTGRNFAAGMSG
          	*** *:***************************************:**************

MG07187.1 	GIAYILDIHHDFMSKLNMEMVEASGLED--PEEIAYVRGLIEDHHHYTGSELAARILVDF
NCU01744.1	GIAYILDIQGDFLTKLNTEMVEASTLEEAEPEEIAFIRSLVEDHHHYTGSELAARILVDF
FG01433.1 	GIAYVLDVHGDFHSKLNGEMVETSGLED--PAEIAFVRGLIEDHHHYTGSERAARILVDF
AN5134.1  	GIAYILDMNQDFLSKVNMEMVECSGLED--PAEIAFLRGLIEDHHHYTGSELAARILLDF
          	****:**:: ** :*:* **** * **:  * ***::*.*:********** *****:**

MG07187.1 	NRALPRFIKVLPVDYKRVLQEEAAK----AAEAKRAEYSLPVLPSHQHKKEE-------K
NCU01744.1	SRALKRFIKVLPVDYKRVLEEEKAKAVKQAAETKRAEYSLPAAA--RKEKDP-------H
FG01433.1 	NRALPRFVKILPVDYKRVLLEEAAK----AAEAKRAEYNLPAVSGVQHKKSE-------K
AN5134.1  	TRALPHFIKVLPTDYKRTLEEEAAK----AAAAKKAETTIPQLPSVPVEKQKPKAEGDAK
          	.*** :*:*:**.****.* ** **    ** :*:** .:*  ..   :*. ..:...::

MG07187.1 	AAKLQDIEETIGDAAADKKK-ALKLDKTKGFMKYHRQNEKYRSSKTRTKDWAEISNRLDE
NCU01744.1	APKLQDIEESITDNAISKKK-ALVLDKTRGFMKYQRRAEKYRKPATRVKDWAELSQRLDE
FG01433.1 	AAKLQDLEEAVGDNAAEKKR-ALVLDKTRGFKMYKRRQEKYRPVNSRLKDWAELSSRLDE
AN5134.1  	KAELLDIEDSVTDSKVEKKRTALILDKTRGFMKYNRRSEKYRNPATRTRDWAELSSRLTE
          	 .:* *:*::: *   .**::** ****:**  *:*: ****   :* :****:*.** *

MG07187.1 	DQLKYQTARCMDCGVPFCQSDSGCPISNIIPKWNELVFS0GQWRDALNRLLMTNNFPEFT
NCU01744.1	DELKYQSARCMDCGVPFCQSDSGCPISNIIPKWNELVFQ~GQWRDALNRLLMTNNFPEFT
FG01433.1 	DELKYQSARCMDCGVPFCQSETGCPISNIIPKWNELVFQ~NQWKDALNRLLMTNNFPEFT
AN5134.1  	DELKYQSARCMDCGVPFCQSDTGCPISNIIPKWNELVFQ~NQWQDALNRLLMTNNFPEFT
          	*:****:*************::****************. .**:****************

MG07187.1 	GRVCPAPCEGACVLGINEDAVGIKSIECAIIDRGFEKGWMVPQPPKVRTGKTVAIIGSGP
NCU01744.1	GRVCPAPCEGACVLGINEQPVGIKSIECAIIDRGFEMGWMVPEPPKIRTGKNVAIIGSGP
FG01433.1 	GRVCPAPCEGACVLGINEDPVGIKSIECAIIDRGFEKGWMVPQPPKVRTGKTVAIIGSGP
AN5134.1  	GRVCPAPCEGACVLGINEDPVGIKSIECAIIDRGFEMGWMVPRPPPVRTGKTVAIIGSGP
          	******************:.**************** *****.** :****.********

MG07187.1 	AGLAAADQLNKAGHLVTVYERSDRLGGLLMYGIPNMKLDKRIVERRTNFMAAEGIQFKTG
NCU01744.1	AGLAAADQLNKAGHNVTVYERADRLGGLLMYGIPNMKLDKRIVKRRTDFMAAEGVQFKTG
FG01433.1 	AGLAAADQLNRAGHLVTVYERADRLGGLLMYGIPNMKLDKRIVKRRTDFMADEGIIFKTG
AN5134.1  	AGLAAADQLNRVGHSVTVYERADRIGGLLMYGIPNMKLDKKIVQRRVDLMAAEGVKFVTG
          	**********:.** ******:**:***************:**:**.::** **: * **

MG07187.1 	VDVGN-GDVTLSSLKATHDAVVICTGATVARDLPIKGRNLEGVHFAMEFLHKNTKSLLDS
NCU01744.1	VAIGE--DITLVDLKKQNDAVIIATGATVARDLPIKNRNLEGIHFAMEFLHKNTKSLLDS
FG01433.1 	VAVGEEGHPSLSDLRASHNVVVIATGATVARDLPIKGRQLEGIHYAMEFLHKNTKSLLDS
AN5134.1  	VSVGPDSEVSLDSLRKSNDAVIIATGATVARDLKVPGRELEGIHFAMQFLHKNTKSLLDS
          	* :* ... :* .*:  ::.*:*.********* : .*:***:*:**:************

MG07187.1 	ELADGSYISAKDKNVVVIGGGDTGNDCIGTSVRHGAKSVINFELLPQPPPERANDNPWPQ
NCU01744.1	ELADGSYISAKDKHVIVIGGGDTGNDCIGTSVRHGAKSVINFELLPKPPPARGRDNPWPQ
FG01433.1 	ELGDNAYISAKDKHVVVIGGGDTGNDCIGTSLRHGAKSVTNFELLPQPPPERANDNPWPQ
AN5134.1  	ELADGAYISAKDKHVVVIGGGDTGNDCIGTSVRHGAKSVVNFELLPQPPPERARDNPWPQ
          	**.*.:*******:*:***************:******* ******:*** *..******

MG07187.1 	WPRIYRVDYGHSEVRQHMGKDPREYCIMSEEFVDDGSGKVKGINTVRVEWTKSASGGWDM
NCU01744.1	WPRVYRVDYGHDEVKQHTGKDPREYCIMSEEFVDDGNGKVRGINTIRVEWTKSASGGWDM
FG01433.1 	WPRIYRVDYGHTEVRQHTGKDPREYCIMSEEFMDDGSGKVKGINTIRVEWTKSPSGGWDM
AN5134.1  	WPRIYRVDYGHSEVKTHMGKDPREYCIMSKEFVDDGNGRVKGINTVRVEWTKSATGGWDM
          	***:******* **: * ***********:**:***.*:*:****:*******.:*****

MG07187.1 	KKVEGSQQFFPADLVLLSMGFLGPEARVLGDDIEKDARKNVKTAPGKYATNMEGVFAAGD
NCU01744.1	KKIEGSQQFFPADLVLLSMGFLGPEARVLGDEIEKDPRKNVKTPPGKYSTNVEGVFAAGD
FG01433.1 	KKVEGSQQFFPADLVLLAMGFLGPEARVLGDEIEKDARKNVKTAPGKYSTNLEGVFAAGD
AN5134.1  	KTVEGSEQFFPADLVLLSMGFLGPEDRLLGEEIERDARKNVKTPPGHYGTNVPGVYAAGD
          	*.:***:**********:******* *:**::**:*.******.**:*.**: **:****

MG07187.1 	CRRGQSLIVW2GINEGRQAAREIDLYLEKYT-SLPVTGGIVKRTAQEIFSAVKPVAPVAA
NCU01744.1	CRRGQSLIVW~GINEGRQAAREVDLYLEKST-CLPVTGGIVKRTAEEVFAAAAAA-----
FG01433.1 	ARRGQSLIVW2GINEGRQAAREIDLYLEKYT-NLPVTGGITKRTAQEIFSQIQVEA----
AN5134.1  	CRRGQSLIVW~GINEGRQCAREVDTFLSGISSQLPVTGGIVRRPAVDAVRQATETVTIAA
          	.********* *******.***:* :*.  :: *******.:*.* : .       . ::

MG07187.1 	
FG01433.1 	
NCU01744.1	
AN5134.1  	
          	
```
